# Phytoestrogens for the Management of Endometriosis: Findings and Issues

**DOI:** 10.3390/ph14060569

**Published:** 2021-06-14

**Authors:** Xia Cai, Min Liu, Bing Zhang, Shao-Jie Zhao, Shi-Wen Jiang

**Affiliations:** 1 Department of Nursing, The Affiliated Wuxi Matemity and Child Health Care Hospital of Nanjing Medical University, Wuxi 214002, China; yzyiheng@163.com (X.C.); liumin_76@163.com (M.L.); 2 Department of Gynecology, The Affiliated Wuxi Matemity and Child Health Care Hospital of Nanjing Medical University, Wuxi 214002, China; yanghezhangbing@163.com; 3 Center of Reproductive Medicine, State Key Laboratory of Reproductive Medicine, Research Institute for Reproductive Health and Genetic Diseases, The Affiliated Wuxi Matemity and Child Health Care Hospital of Nanjing Medical University, Wuxi 214002, China

**Keywords:** phytoestrogens, endometriosis, resveratrol, isoflavones, puerarin

## Abstract

Endometriosis, a chronic disease characterized by recurrent pelvic pain and infertility, severely impacts the health and life quality of many women worldwide. Since phytoestrogens are commonly found in a variety of foods, and estrogen is a major pathological factor for the pathogenesis of endometriosis, their possible involvement cannot be ignored. This review summarizes data on the relationship between phytoestrogen intake and endometriosis risk, and analyzes the findings from in vitro experiments, rodent endometriotic models, and human intervention trials. While favorable results were often obtained from endometrial primary cultures and animal models for resveratrol, isoflavones and puerarin, only resveratrol showed promising results in human intervention trials. Critical issues concerning the current study efforts are discussed: the possible reasons beneath the discrepant observations of estrogenic/anti-estrogenic effects by phytoestrogens; the complicated interplays between phytoestrogens and endogenous estrogens; the shortage of currently used animal models; the necessity to apply reasonable doses of phytoestrogens in experiments. It is expected that the analyses would help to more properly assess the phytoestrogens’ effects on the endometriosis pathogenesis and their potential values for preventive or therapeutic applications.

## 1. Introduction

Endometriosis refers to the ectopic implantation and growth of endometrial tissues frequently found in the ovary, ligaments, peritoneal surface, bowel and bladder [1]. This disease, clinically manifested as chronic pelvic pain and infertility [1,2], affects as many as 6% to 10% of reproductive women [1,3,4]. Endometriosis patients are currently treated with hormonal suppression of active disease, symptomatic management, and surgical ablation of visible lesions [5]. These therapeutic procedures cannot overcome the high recurrent propensity of this disease [6], and in many cases the disease becomes a lingering illness negatively impacting the fertility and life quality. Long-term application of natural compounds, e.g., phytoestrogens from diet sources, has been investigated for their potential preventive and therapeutic benefits [7].

Several theories concerning the physio-pathogenic process of endometriosis are raised and studied by investigators [5]. The retrograde menstruation hypothesis emphasizes on the ectopic growth of endometrial tissues regurgitated during menstrual cycles. It was proposed that in this process the endometrial stem cells may play a key role. Coelomic metaplasia and localized inflammatory responses may explain the rise of peritoneal lesions. Vascular and lymphatic metastasis could be the origin of extrapelvic lesions. On the cellular and molecular levels, evidences for excessive estrogen stimulation, inflammation, increased angiogenesis, and active proliferation are commonly observed in endometriosis cases. Among these changes, excessive estrogenic activity is well-recognized for its extensively involved in all the pathological aspects of endometriosis.

Although the endometriotic tissues are not malignant, their ectopic establishment, invasive growth and structural damages to adjacent tissues afford it certain resemblance to the characters of malignant tumors. In addition, a meta-analysis by Kim et al. covering more than 400,000 endometriosis cases showed that endometriosis was associated with an increased risk of endometrial cancer [8]. An increased risk of ovarian cancer in patients with ovary endometriosis was also reported [9]. Kajiyama et al. pointed out that endometriosis could be a precursor lesion of epithelial ovarian carcinomas, and may exert a tumor-promoting effect on the extra-ovarian cancers [10]. Local estrogen production by endometriotic tissues may lead to malignant transformation of ovarian epithelium through the paracrine mechanism.

The very different prevalence of endometriosis among various regions/countries and populations seems to suggest that life style, exposure to certain environmental factors such as sun exposure and especially diet preference, are important etiological factors in addition to genetic backgrounds [11,12]. Diet adjustment and herb supplements are discussed among patients and clinicians for symptom management. Gluten-free diets could improve the symptoms in some women who have endometriosis and gastrointestinal complains [13]. Many types of foods, mostly soybeans, soy-related products, grapes, and oriental herbs are rich in phytoestrogens. Phytoestrogens can bind to estrogen receptor (ER) and affect ER-mediated responses. Numerous in vitro and epidemiological studies have been performed to delineate the relationship between phytoestrogens and endometriosis. Resveratrol and other phytoestrogens have been investigated for potential benefits in endometriosis patients. In spite of these study efforts, important issues regarding the etiological and therapeutic/preventive effects of phytoestrogens on endometriosis are far from a settlement. This review analyzes the available data on the effects and mechanisms of phytoestrogens in endometriosis, discusses the methodological loopholes and potential bias in relevant studies, and provides comments on several critical issues.

The authors applied the following criteria and methods for material collection and review: (1) Various combinations of these keywords were applied for literature search using the PubMed database: endometriosis/endometriotic; ectopic/eutopic endometrium; phytoestrogen; estrogen; ER/estrogen receptor. (2) When searching data related to a specific phytoestrogen, resveratrol, isoflavones, puerarin and other names of individual compound were used. (3) No limit on publication time, journal, country, or language was applied during literature search. (4) For all cited references, full-length articles were obtained, reviewed and discussed among authors. (5) For both in vitro and in vivo studies, whenever available, detailed information on the doses of phytoestrogens and study models were collected and documented. (6) Original studies with proper experimental design and carrying critical information were listed as key references.

## 2. Estrogen as a Dominant Pathogenic Factor for Endometriosis 

In spite of intensive studies, many aspects of the pathogenic mechanism of endometriosis remains controversial. Multiple molecular and cellular events, including epigenetic changes, stem cell trafficking and alterations of natural killer T may contribute to the development of endometriosis [14,15]. Among many studies on different events, pathways, and theories, accumulated data indicated that an increase in estrogen levels may play a significant role for the pathogenesis of endometriosis [3]. While the circulatory estrogens produced by ovary, adipose and muscle can reach endometrium, the actions of locally biosynthesized estrogens and paracrine mechanism have been emphasized by some investigators. Smuc et al. and Mori et al. noted that in spite of stable circulatory estrogen levels in endometriosis patients, the estrogen levels in menstrual blood of these patients significantly increased in comparison to healthy controls [16,17]. Huhtinen et al. determined the estrone (E1) and estradiol (E2) levels in the ectopic endometrium from the surface of ovary and peritoneal membranes using the method of liquid chromatography–tandem mass spectrometry [18]. The results showed that the estrogen levels in ectopic endometrium were significantly higher than those in eutopic endometrium from either endometriosis patients themselves or unrelated healthy women. The estrogen levels in the ectopic endometrial tissues from peritoneal surface or deep invasion did not change during menstrual cycles [18], pointing to a constant nature of local estrogen biosynthesis. It was reported that the mRNA and protein levels of steroidogenic acute regulatory (StAR) and aromatase, the key enzymes for estrogen biosynthesis, were elevated in the ectopic endometriotic tissues [19], indicating an active local estrogen synthesis. These findings highlight the significance of locally synthesized estrogens for a sustained progression of endometriosis in many patients.

Accumulated data supports that estrogen is able to promote the survival, migration, adhesion, and proliferation, of endometrial epithelial and stromal cells. Xu et al. found that primary cultures from eutopic endometrial tissues of infertile endometriosis women expressed higher levels of H19 and ACTA2 (alpha smooth muscle actin, α-SMA) than those from infertile, non-endometriosis women, and the alterations in the estrogen/H19/miR-216a-5p/ACTA2 pathway may contribute to the invasion and migration of the epithelial cells [20]. Qi et al. reported that eutopic epithelial cells isolated from endometriosis patients had increased expression of epithelial-mesenchymal transition-related genes such as Notch1, Slug, Snail and N-cadherin, and the blocker of epithelial-mesenchymal transition inhibited the 17β-estradiol (E2)-induced cell proliferation, migration and invasion [21].

Both ER isoforms, ERα and ERβ, are constitutively expressed in the human endometrium, and ERβ may play a unique regulatory role(s) for endometrial physiology. It was reported that in endometriotic lesions there were relatively higher levels of ERβ and altered ERα/ERβ ratios [22,23]. These changes might lead to the deregulation of target genes such as Greb-1 and c-Myc, resulting in inflammation and endometriosis progression [2,3,24]. In cultured endometriotic stromal cells, the ERβ levels are 142-fold higher, and ERα levels are 9-fold lower, than those in the normal endometrium [25]. Chantalat et al. investigated the functions of ERs for the regulation of endometrium, and found that overexpression of ERβ in stromal cells inhibited the tumor necrosis factor alpha (TNF-α)-mediated apoptosis, suppressed the expression of ERα, and induced interleukin-1 production [3]. In uterine microvascular endothelial cells, ERβ was able to mediate the E2-stimulated cyclooxygenase (COX) expression and prostaglandin E2 (PGE2) production [26], again pointing to the significance of ERβ for the inflammatory responses in the endometriotic tissues.

Inflammation largely accounts for the pain and infertility suffered by endometriosis patients [2,27]. Macrophage infiltration and elevated inflammatory factors such as Interleukin 6 (IL-6) can be found in the endometriotic tissues, adjacent tissues, and peritoneal fluid [28]. COX-2 is known to mediate a variety of inflammatory responses in asthma, acute lung injury, idiopathic pulmonary fibrosis (IPF) and inflammatory renal disease [29,30]. Studies have shown that the expression of COX-2 in the eutopic as well as ectopic endometrium of endometriosis patients was significantly higher than that in the normal endometrium of healthy women [31,32]. The increased COX-2 expression could lead to an elevated production of PGE2, an overexpression of aromatase P450 and increased estrogen synthesis. The positive feedback loop of “E2-COX-2-PGE2-aromatase-E2” could form a vicious circle between chronic inflammation and estrogen production [33], to support the survival and proliferation of ectopic endometrial tissues.

## 3. Phytoestrogens from Food Consumption 

### 3.1. Categories and Contents of Phytoestrogens from Diet Sources 

The generic name phytoestrogen refers to a group of nonsteroidal phenolic plant compounds such as isoflavones, lignans, coumestans that broadly found in a variety of vegetables, fruits, grains, and especially soybeans and related products such as bean curds/tofu and soy milk [34,35]. The European Prospective Investigation into Cancer and Nutrition (EPIC) is a multi-center cohort study focusing on the diet, environment and life style in European countries [36,37]. According to data from this study, the total intake, subcategories of phytoestrogens as well as their food origins varied widely among different countries/regions [38]. In England, the average intake of health-conscious population reaches 24.9 and 21.1 mg/d for males and females, respectively. But in males of Greece and females of Granada region of Spain, the average intake drops to 1.3 and 1.0 mg/d, respectively. The vast variation in the phytoestrogen intake could be true in a worldwide scope. It is noteworthy that in the East Asian countries like China, Japan and Korea, the phytoestrogen intake could be much higher due to the more frequent consumption of soybean products [12,39]. In addition, traditional medicines are popular in these countries, and some herbs contain high levels of puerarin and other phytoestrogens. It was noted that soybean produced in different regions or at different years could contain varied quantities of phytoestrogens [40], which raised a challenge for the intake assessment using questionnaire-based methods.

### 3.2. Bioavailability of Phytoestrogens

Most phytoestrogens in foods exist in an inactive glycoside conjugate form. After ingestion, these molecules will be hydrolyzed by bacterial β-glucosidase to release aglycones as primary metabolites in the small intestines and colon. Aglycones and the secondary metabolites transformed by colonic microflora will be absorbed or excreted [41]. A wide range of phytoestrogen bioavailability is found among individuals. Arai et al. conducted a 3-day survey of 106 volunteers (29–78 years old) from north Japan, and recorded an intake range from 18.2 to 167.2 μmol/day, and from 30.6 to 282.8 μmol/day, for daidzein and genistein, respectively [42]. The serum levels of daidzein and genistein ranged broadly from 3.0 to 1766.2 nmol/L and from 25.3 to 2459.7 nmol/L, with the urine levels ranged from 0.2 to 155.2 μmol/day and from 0.2 to 62.4 μmol/day, respectively. Nonetheless, for each compound, close correlations were found between the dietary intake and plasma concentration, and between the dietary intake and urinary excretion. The relative bioavailability could be affected by gender, the intestinal transit time and intestinal flora [43,44,45]. As noted by some investigators, these variations may contribute to the discrepant results seen in different clinical studies [46].

### 3.3. Bioactivities of Phytoestrogens

Available data indicate that most phytoestrogen activities are mediated by ERs. By their structural similarity to estrogens, these compounds can bind to both ERα and ERβ, but with much lower binding affinities than estrogens. Kuiper et al. systematically measured the affinity of E2, coumestrol, genistein, daidzein, biochanin A and formononetin with ERs using the competition binding assay. It was found that the ER-binding affinities of phytoestrogens were at least three orders lower than that of E2 [47]. Interestingly, while E2 has a similar affinity to ERα and ERβ, phytoestrogens show a relatively higher affinity to ERβ than to ERα and display a stronger competition with E2 in binding to ERβ than to ERα. Kostelac et al. observed that in the absence of E2, ERα and ERβ were able to interact with estrogen response element (ERE) immobilized on the surface of a sensor chip [48]. Upon binding to E2 the ER affinity to ERE was increased by 50%. In addition, when binding to different phytoestrogens, ER molecules displayed diverged efficacies to interact with immobilized ERE, from high to low in terms of effective concentration (EC50): For ERα, E2 (0.03 μmol/L) > coumestrol (0.2 μmol/L) > equol (3.5 μmol/L) > genistein (15 μmol/L) > daidzein (>300 μmol/L); for ERβ, E2 (0.01 μmol/L) > coumestrol (0.025 μmol/L) > genistein (0.03 μmol/L) > daidzein(0.35 μmol/L) > equol (0.4 μmol/L). Thus, the ratios of EC_50_α/EC_50_β for each compound were: E2, 3; coumestrol, 8; equol, 8.8; genistein, 500; daidzein > 850. This order of efficacy indicated that genistein and daidzein significantly prefer binding to, and activation of, ERβ than ERα. Křížová et al. confirmed the phytoestrogens’ differential affinities to ERs [49]. For example, isoflavones bound to ERβ with a 5-times greater affinity than to ERα, suggesting that ERβ could be more important than ERα as far as the phytoestrogen bioactivity was concerned.

Studies have demonstrated the potency of phytoestrogens for transcriptional activation. In the above described study by Kuiper et al., the estrogenic activities of phytoestrogens were determined with a transient overexpression of recombinant human ERα or ERβ in 293 human embryonal kidney cells [47]. It was found that genistein, coumestrol and zearalenone stimulated the transcriptional activity of both ERα and ERβ at a concentration range of 1–10 nmol/L. Although the functional concentration of phytoestrogen in this experiment was comparable with the serum E2 concentration of reproductive women, most circulatory E2 bound to SHBG protein, with only less than one tenth of E2 being bioactive. The study also established a rank of the estrogenic potency of E2 and phytoestrogens with the transactivation assay: for ERα, E2 >> coumestrol > genistein > daidzein > biochanin A > formononetin; for ERβ, E2 >> genistein = coumestrol > daidzein > biochanin A > formononetin. Another study by Morito et al. revealed that while the binding efficiency of genistein to ER was almost the same as that of E2, the concentration required for genistein to induce transcription was 10^4^ times that of 17β-estradiol [50]. It was speculated that the ER structural transformation upon genistein binding may not be sufficient to promote the subsequent interaction with co-activators.

Although estrogen is known to be capable of affecting cell functions through ER-independent pathways [51], it is unclear if phytoestrogens may have similar activities. Scherbakov et al. observed that a high dose (50 μmol/L) of apigenin, genistein, quercetin or naringenin showed comparable apoptotic effects in ER^+^ and ER^−^ breast cancer cell lines [52], suggesting that the cytotoxic effects were not mediated by ER. Interestingly, using a cell-free assay, Edmunds et al. observed that naringenin and chrysin were able to directly inhibit the activity of human recombinant aromatase [53], demonstrating an ER-independent mechanism of phytoestrogens. It should be mentioned that besides their direct actions in peripheral organs, phytoestrogens can indirectly affect peripheral organs through regulating the hypothalamus-pituitary-gonad axis. Arispe et al. reported that in vitro treatment of ovine pituitary cells with coumestrol, zearalenone and genistein for 48 h led to a significant suppression of basal secretion of the follicle-stimulating hormone (FSH) [54]. Addition of the ER blocker ICI 182 780 resulted in reversal of the suppression, suggesting the involvement of an ER-mediated mechanism. Through the neuroendocrine system, phytoestrogens’ effects would be amplified and/or expanded to diversified organs and tissues, to affect even the pathophysiological processes not strictly estrogen-dependent.

## 4. Uncertain Relationship between Phytoestrogen Intake and Endometriosis Risk

Multiple case-control studies were conducted to investigate the relationship between phytoestrogen intake and endometriosis risk. Youseflu et al. compared the phytoestrogen intake of 78 women (15–45 years old) with laparoscopically confirmed endometriosis versus 78 normal controls (15–45 years old) [55]. Data on the consumption of 147 types of phytoestrogen-containing foods was collected for the past year by trained dietitians using a questionnaire, and converted to the daily intake. The phytoestrogen intake was estimated using the United States Department of Agriculture database for phytoestrogen contents in each type of food. The calculation showed that among many phytoestrogens, the intake levels of isoflavones, lignan, and coumestrol through consumption of dairy products and coumestrol from fruits were inversely correlated with the risk of endometriosis.

Tsuchiya et al. measured the urine levels of genistein and daidzein in 138 premenopausal women suffering infertility [56]. The endometriosis group included 79 patients with early (Stage I–II) or advanced (Stage III–IV) endometriosis, and the control group included 59 women without endometriosis diagnosis. The urine genistein and daidzein levels, respectively, were found to be inversely associated with the risk of advanced endometriosis, but not with early stage endometriosis. These observations appeared to suggest that genistein and daidzein might suppress the progression rather than occurrence of endometriosis. However, since infertility is known to be associated with endocrine dysregulation as well as endometrial abnormality, these confounding factors may cause some uncertainty in data interpretation.

While the above reports are in favor of a benefit of phytoestrogen, a case report seemed to suggest adverse effects of phytoestrogens on endometrium [57]. Chandrareddy et al. observed that among three women with high soy product consumption, one displayed postmenopausal bleeding with uterine polyps, a proliferative endometrium, and a progressing leiomyoma; one presented endometriosis, severe dysmenorrhea, abnormal uterine bleeding, and uterine leiomyoma; another suffered endometriosis, severe dysmenorrhea, abnormal uterine bleeding, and uterine leiomyomata, and secondary infertility. In addition, when these women withdrew soy from the diet, their endometrial symptoms were alleviated.

Yet other studies did not observe a clear effect of phytoestrogen intake on endometriosis. Mumford et al. measured the urinary levels of genistein, daidzein, O-desmethylangolensin, equol, enterodiol and enterolactone, and conducted two comparisons in an age-matched manner [58]. The first comparison was between two groups of women who received laparoscopy or laparotomy. One group included 189 women with endometriosis and another included 283 women without endometriosis. The second comparison was carried out between two groups of women without operation. One group included 14 women with endometriosis, and another included 113 women without endometriosis. Neither comparison found a significant difference in the urinary concentrations of selected phytoestrogens between the groups with and without endometriosis [58].

Considering the study method, a questionnaire-based estimation of intake may suffer a disadvantage of information inaccuracy [59,60]. On the other hand, a direct measurement of urinary phytoestrogens only transiently reflects the short-term rather than long-term intake levels [43]. Moreover, as discussed later, women with different endogenous estrogen levels may respond differentially to phytoestrogens. These methodological dilemmas, together with the complicated interplays between endogenous estrogens and phytoestrogens as well as the small subject sizes may contribute to the above described discrepancies. A simultaneous intake recording and urine phytoestrogen measurement may help to establish a quantitative correlation, either linear or non-linear, between the intake levels and urinary concentrations. Further studies using objective parameters and larger cohorts are required to clarify if phytoestrogen could be a risk or beneficial factor for endometriosis.

## 5. Phytoestrogens and Endometriosis

### 5.1. Resveratrol

#### 5.1.1. Findings from In Vitro Studies

Resveratrol is a natural phytoalexin polyphenol compound broadly existing in grapes, wines, berries and nuts [28,61]. Wine and the skin of grape are rich in resveratrol, reaching levels of 1.52 mg/L and 50–100 μg/g, respectively [62]. Beneficial effects of resveratrol including anti-cancer, anti-oxidative, anti-inflammatory, anti-organisms, anti-arteriosclerotic, and anti-angiogenic activities have been reported [28,63]. For endometriosis studies, Arablou et al. determined the effects of resveratrol in the endometrial stromal cells isolated from eutopic and ectopic tissues of endometriosis patients, and from endometrial tissues of healthy controls [64]. The results indicated that treatment with 100 μmol/L of resveratrol led to a decreased expression of insulin-like growth factor-1 and hepatocyte growth factor in the cells from all three types of tissues, with the highest potency observed in the cells from patients’ ectopic tissues. Similarly, Kolahdouz-Mohammadi et al. reported that resveratrol at the same concentration as above was able to inhibit the expression of monocyte chemotactic protein-1 (MCP-1), IL-6 and IL-8 in the primary cultures of endometrial stromal cells and ectopic tissues [7]. Villanueva et al. examined the functional interaction between resveratrol and the low density lipoprotein cholesterol (LDLC) reduction reagent simvastatin in human endometrial stromal cells isolated from healthy volunteers [65]. It was observed that resveratrol potentiated the inhibitory effects of simvastatin on cholesterol biosynthesis and the enzyme activity of the 3-hydroxy-3-methylglutaryl-coenzyme A reductase (HMGCR). Moreover, resveratrol abrogated the stimulatory effects of simvastatin on HMGCR mRNA as well as protein expression. All these results support that resveratrol alone or in combination with other reagents may be useful for treatment of endometriosis.

#### 5.1.2. Findings from Rodent Study Models

At least five studies on resveratrol were conducted in mouse endometriotic models created by heterotransplantation of human endometrial tissues, autotransplanted mouse endometrial tissues, or allotransplanted mouse endometrial tissues. Bruner-Tran et al. administered resveratrol (6 mg/day) and E2 (8 mg/day, silastic capsule) through gavage to an endometriotic mouse model generated by ophorectomization and intraperitoneal injection of human endometrial tissues [66]. Compared with the E2 group, the resveratrol group exhibited a reduced number and size of endometriotic implants; Amaya et al. examined the resveratrol effects on the expression of ERα, Ki-67 (a proliferative marker), aryl hydrocarbon receptor, and cytochrome P450 super family members CYP1A1/CYP1B1, in human endometrial tissues peritoneally transplanted to the ovariectomized immune deficient mice [67]. After 30 days of treatment with subcutaneous embedding of slow releasing pellets, a steady concentration of 6 mg/day resveratrol displayed an agonist, whereas 60 mg/day of resveratrol exhibited an antagonist effect on the E2-mediated gene expression; Rudzitis-Auth et al. used an alternative model formed by allotransplantation of uterine tissues from donor mice to the abdominal wall and mesentery of recipient mice [68]. After 4 weeks of resveratrol (40 mg/kg/day) oral gavage, the angiogenesis in peritoneal and mesenteric endometriotic lesions, as measured by the microvessel density, was significantly reduced. Immunohistochemical analyses indicated a decreased proliferation of vascular endothelial cells in the endometriotic lesions. In resveratrol-treated mice the endometriotic lesions exhibited a decreased growth rate and final size, which is related to a reduced numbers of proliferating cell nuclear antigen (PCNA)- and Ki-67-positive stromal and glandular cells; Ricci et al. reported two lines of evidence in support of the therapeutic effects of resveratrol and epigallocatechin-3-gallate (EGCG) [69]. In primary culture of eutopic endometrial epithelial cell cultures isolated from women at the proliferative phase, both resveratrol (25–100 μmol/L) and EGCG (20–100 μmol/L) significantly reduced the cell proliferation and increased the cell apoptosis. In a mouse autotransplant model, continual peritoneal injection of resveratrol (10 and 25 mg/kg/day) or EGCG (20 and 100 mg/kg/day), started at 2-week post-surgery and lasted for 4 weeks, was able to reduce the number and volume of endometriotic lesions, cell proliferation and vascular density, but to increase cell apoptosis, within the lesions; Kong et al. reported that in a mouse autotransplant model, daily peritoneal injection of 25 mg/kg for 4 weeks inhibited the expression of metastasis-associated protein 1 [70], a gene product known to promote endometrial stromal cell proliferation, migration, and invasion by inducing the epithelial-mesenchymal transition. Thus, studies in mouse models uniformly demonstrated a beneficial effect of resveratrol in endometriosis-like lesions.

At least 5 studies on resveratrol were performed using the rat autotransplant model. Yavuz et al. observed that resveratrol (1 mg/kg/day and 10 mg/kg/day) intraperitoneal injection for 7 days was able to reduce the volumes as well as the histological scores of the implants [71]; Ergenoğlu et al. observed that following muscle injection of resveratrol (10 mg/kg/day) for 14 days inhibited the vascular endothelial growth factor (VEGF) expression in the peritoneal fluid/plasma and MCP-1 expression in the peritoneal fluid, and reduced the size of implant [72]; Tekin et al. observed that muscle injection of resveratrol (30 mg/kg/day) for 14 days reduced the volumes, histopathological grades, and VEGF expression in the implants, and IL-6, IL-8 and TNF-α in the peritoneal fluid [73]; Cenksoy et al. reported that oral administration of resveratrol (60 mg/kg/day) for 21 days led to reduced lesion areas, histopathological grades, and VEGF staining scores of the implants, and reduced VEGF and MCP-1 levels in peritoneal fluid [74]; Bahrami et al. investigated the therapeutic effects of resveratrol and the LDL reduction reagent atorvastatin on the expression of glucose transporters 1 and 3 (GLUT-1 and GLUT-3), monocarboxylate transporters 1 and 4, and neovascularization in the implants [75]. The results showed that resveratrol (40 mg/kg/day) for 28 days alone or in combination with atorvastatin were able to reduce the size and vascularization as well as the expression of tested genes in the implants. These results from rat models consistently suggested that resveratrol when administered either orally or by muscle/peritoneal injection could inhibit the growth as well as the expression of key inflammatory and angiogenic factors in the implants.

#### 5.1.3. Findings from Human Population

For studies in human subjects, Maia et al. assessed the pain scores following oral administration of contraceptives (drospirenone/ethinylestradiol, 3 mg/30 μg) alone or in combination with resveratrol (30 mg/day) in 12 patients with endometriosis and dysmenorrhea [76]. While the contraceptives failed to show a benefit, the addition of resveratrol to contraceptives for two months resulted in a significant reduction in pain scores, with 82% of patients reporting complete resolution of dysmenorrhea and pelvic pain. In a separate comparison, a significantly greater inhibition of aromatase and COX-2 expression in the eutopic endometrium was observed in the contraceptives + resveratrol group (26 patients) compared to the contraceptives alone group (16 patients); However, in a randomized clinical trial conducted by Mendes da Silva et al. in 44 endometriotic women, no additional benefit of resveratrol (40 mg/day) was observed when combined with the monophasic contraceptive (levonorgestrel 0.15 mg/ethinylestradiol 0.03 mg) for the treatment of endometriosis-related symptoms [77]; Kodarahmian et al. investigated the effects of resveratrol on the mRNA and protein expression levels of matrix metalloproteinase-2 and -9 (MMP-2 and -9) in the eutopic endometrium at mid-secretory phase [78]. Thirty four 18–37 years old women with severe endometriosis (stage III-IV) were randomly divided into the treatment (N = 17) and control (N = 17) groups. The results from biopsy samples showed that intervention with resveratrol (400 mg, twice a day) for 12–14 weeks led to a significant decrease of these genes’ expression. The same group applying the same experimental design and doses, most likely using the same set of samples, subsequently found that intervention with resveratrol resulted in decreased VEGF and TNF-α mRNA as well as protein expression levels in the eutopic endometrium [79]. Thus, the majority of clinical studies tend to support that resveratrol can relieve the endometriotic symptoms by inhibiting the expression of key angiogenic and inflammatory factors in endometriotic tissues.

Overall, the positive data from multiple in vitro experiments and animal studies repeatedly point to the potential value of resveratrol for therapeutic use. Several human studies showed that resveratrol could be beneficial for the management of endometriosis. Randomized clinical trials with larger scale are required to prove the therapeutic value of this phytoestrogen. In addition, safety issues, especially those concerning the collateral negative effects in non-endometrial organs, remain to be addressed.

### 5.2. Isoflavones

Isoflavones form a subclass of phytoestrogen including at least 12 types of phenolic compounds [80]. Soybeans, isolated soy proteins, soymilk and fermented soybean foods such as tofu, natto and soy sauce represent the main dietary sources of isoflavones [81]. Sugar conjugates of genistein, daidzein and glycitein compose 90% of the total isoflavone intake from soybean-derived foods [80]. Extensive studies demonstrated that isoflavones may have various health benefits, mostly for ameliorating the negative impacts of menopause on cardiovascular diseases, osteoporosis and regression of cognitive function [82,83,84].

#### 5.2.1. Findings from In Vitro Studies

Numerous in vitro studies have been performed using the endometrial cancer cell lines or primary cultures of endothelial and stromal cells isolated from endometrium. As discussed later, depending on the experimental system and background estrogen levels, genistein displays either estrogenic or antiestrogenic effects. Edmunds et al. reported that naringenin and chrysin directly inhibited the enzyme activity of human recombinant aromatase in a cell-free assay, but genistein and daidzein did not [53]. However, in primary cultures of human endometrial stromal cells, genistein (1 nmol/L to 1 mmol/L) upregulated the expression of aromatase activity and increase the aromatase activity, suggesting that genistein could modify the natural course of endometriosis thorough affecting local estrogen biosynthesis. Also in endometrial stromal primary cultures, Salsano et al. observed that 10, 20, 50, and 100 μmol/L of genistein, daidzein, or genistein + daidzein inhibited cell proliferation and prolactin secretion, and induced in vitro decidualization, in a dose-dependent manner [85]. Srisomboon et al. demonstrated that in the primary culture of porcine endometrial epithelial cells, genistein and daidzein enhanced β-defensins (PBD)-2 expression and PBD-2 and PBD-3 secretion [86]. While it was thought that this effect could promote the innate host defense of endometrium against infection, its involvement in the endometriosis could not be excluded.

#### 5.2.2. Findings from Rodent Study Models 

Many laboratories have used rodent models to investigate the in vivo effects of isoflavones. Ronis et al. conducted a comparative study on casein, soy protein isolate (SPI), E2, a combination of SPI and E2, and the carcinogen dimethylbenz(a)anthracene in mice. Each mouse was given on the average 19.3 mg/kg/day of genistein and 9.2 mg/kg/day of daidzein, and received oophorectomy at postnatal day 50 [87]. After 35 days of treatment, SPI did not affect the weight of uterus, but transcriptome analysis of the mouse uterine tissues detected 152 genes with altered expression. Cross-checking with the 1991 genes altered in E2 treatment group led to the identification of 67 overlapping genes. Gene function annotation indicated that while E2 upregulated the oncogenic and extracellular matrix pathways, SPI upregulated the peroxisome proliferator activated receptor pathway and fatty acids metabolism. Comparison among groups of E2, SPI and the combination of the two revealed a SPI inhibition of E2-induced uterine proliferation in the presence of dimethylbenz(a)anthracene, as indicated by corresponding changes in PCNA and Ki-67 mRNA levels. The authors concluded that E2 and isoflavones may preferentially regulate different uterine functions. Specifically, SPI could serve as a selective estrogen receptor modulator (SERM), and display an anti-estrogenic activity through co-regulation of estrogen-responsive genes with endogenous estrogens.

Takaoka et al. reported that the dietary supplements isoflavone aglycones (DRIAs) (0.2–20 μmol/L) inhibited the proliferation of primary cultures isolated from ovarian endometrioma, but not primary cultures from normal endometrium [88]. Interestingly, daidzein, genistein, and glycitein did not show such an effect. The dietary supplements-induced inhibition was reversed by an ERβ antagonist, or the siRNA-mediated ERβ knockdown, but not by an ERα antagonist, highlighting the importance of ERβ. Moreover, DRIAs inhibited the expression of IL-6, IL-8, COX-2 and aromatase, the enzyme activity of aromatase, the TNF-α-induced IκB phosphorylation as well as the p65 translocation to nuclei. An ovariectomized mouse endometriosis model was constructed by transplanting the donor-mouse uterine fragments to recipient mouse. The DRIA-containing feeds reduced serum levels of the glucocorticoid-regulated kinase and PGE2, and decreased the number, weight, and Ki-67 proliferative activity of endometriosis-like lesions compared to the lesions from untreated control mice. Similarly, Sutrisno et al. reported that oral administration of genistein at all the tested doses (0.78; 1.04; 1.3 mg/day, 15 days) inhibited the expression of ERα, VEGF and HIF-1α, but increased the expression of ERβ, in the human endometriotic tissues transplanted to mice [89]. Consistent results were reported by Yavuz et al. in the rat autotransplant model [90]. Oral administration of either genistein (500 mg/kg/day) or SERM raloxifene (10 mg/kg/day) reduced the surface areas and the histopathologic scores of the implants. However, Cotroneo et al. observed different results in the ovariectomized rat model. Continual daily injection (3 weeks) of genistein (16.6 and 50 μg/g) or E1 (1 μg/rat), but not dietary genistein (250 or 1000 mg/kg) sustained, rather than inhibited, the growth of uterine tissues autotransplanted to the intestinal mesentery [91]. This study and the study by Sutrisno et al. [89] applied similar doses of genistein, but used human endometriotic tissues and normal mouse endometrial tissues, respectively, which could account for their divergent observations. Nevertheless, most animal model studies showed an inhibitory effect of some isoflavones against the in vivo growth of endometriosis-like implants, possibly through suppressing the expression of key antigenic and inframammary factors. 

#### 5.2.3. Findings from Human Population

Duncan et al. examined the effects of isoflavones in 18 postmenopausal women [92]. The results showed that oral administration of soy powders containing different levels (7.1 ± 1.1, 65 ± 11, 132 ± 22 mg/d) of isoflavones for 93 days had no significant influence on the plasma levels of estrogen, androgen, gonadotropin, sex hormone binding globulin (SHBG), prolactin, insulin, cortisol, thyroid hormone, the vaginal cytology and endometrial biopsy. Detailed data analyses showed a small but statistically significant decrease in estrone-sulfate, a trend toward a decrease of E2 and E1, and a small but significant increase in SHBG, in the high-isoflavone diet group. For other hormones including estrogen, androgen, gonadotropin, prolactin, insulin, cortisol and thyroid hormone, the changes were all too small to have a physiological importance. Consistently, negative results were also obtained in a 6-month study by Murray et al. [93] and a 3-month study by Hale et al. [94]. Quaas et al. conducted a randomized, double-blinded study with the use of placebo control, in 224 healthy postmenopausal, 45–92 years old women [95]. The results showed that a 3-year isoflavone soy protein supplementation (154 mg) did not cause any significant changes in the endometrial thickness and the incidence of endometrial hyperplasia or endometrial cancer. On the other hand, Unfer et al. [96] reported that the 5-year supplement of isoflavones (150 mg/d) led to an increased incidence of endometrial hyperplasia in healthy postmenopausal women with intact uterus.

Taken together, although experiments using primary endometrial cell cultures and rodent endometriotic models suggested a potential therapeutic value of isoflavones, studies in non-endometriosis women showed no significant impact of isoflavones on the hormonal levels or endometrium biopsies. Instead, an increased risk of endometrial hyperplasia was observed following the long-term supplementation of isoflavones. The effects of isoflavones on endometriotic tissues have not been specifically investigated in human subjects.

### 5.3. Puerarin

Puerarin is a flavonoid compound isolated from the radix of the Chinese herb *Puerarina Lobate*. This compound is used for the treatment of various cardiovascular disorders, alcoholism and neurological diseases. Beneficial effects including cardioprotective, neuroprotective, anti-oxidative and anti-inflammatory actions have been reported [97]. Wang et al. investigated the effects of puerarin and E2 in the stromal cell primary cultures isolated from the ectopic endometrium of premenopausal endometriosis patients [98]. It was found that while E2 increased the expression of MMP-9 and decreased the expression of the tissue inhibitor of metalloproteinase 1 (TIMP-1), promoted the cell invasiveness, and stimulated vascularization, addition of puerarin (10^−9^ mol/L) to E2 (10^−8^ mol/L) was able to reverse these estrogenic activities. Cheng et al. reported that puerarin was able to bind competitively to ER in stromal cells isolated from human ovary endometriotic cysts [99]. In addition, puerarin (10^−9^ mol/L) suppressed the E2 (10^−8^ mol/L)-induced cell proliferation, partly via impeding the rapid, non-genomic, membrane-initiated ERK pathway.

Chen et al. investigated the in vivo effects of puerarin with the use of an endometriosis model created by autotransplanting the uterine tissues to abdominal peritoneum of mature female rats [100]. At 4 weeks post-operation, 60, 200 or 600 mg/kg of puerarin was given daily thorough oral gavage for 4 weeks. The results showed that all the tested doses of puerarin inhibited the proliferation of ectopic endometrium. A suppression on the aromatase expression and reduction of local estrogen biosynthesis was observed at the low level, but not at high levels, of puerarin. The authors speculated that the different effects might be explained by the complicated mechanisms of puerarin, including those related to E2 production and ERα expression. Kim et al. reported that the pueraria flower extracts (25–100 μg/mL) were able to suppress the adhesion of immortalized human endometriotic cells, and reduced the mRNA and protein expression of MMP-2 and MMP-9 [101]. Moreover, puerarin activated the extracellular signal-regulated kinase 1/2 (ERK1/2) pathway, and an ERK1/2 inhibitor abrogated the puerarin-mediated inhibition of cell migration. Furthermore, a mouse endometriotic model was generated by the injection of endometrial tissues from donor mouse to the peritoneal cavity of recipient mouse. Oral administration of puerarin (150 and 300 mg/kg/day) for 5 weeks led to a suppressed formation of endometriosis-like lesions. In another study, Yu et al. found that puerarin (5–80 mg/kg/day) reduced the levels of E2 and PGE2 and inhibited the growth of ectopic endometrial tissues in a rat autotransplant model, likely by inhibiting the expression of aromatase and COX-2, and/or upregulating of ERβ in the ectopic endometrial tissues [102].

Taken together, in vitro experiments using cell cultures indicated that puerarin may affect the expression of key factors involved in the pathogenesis of endometriosis. Several studies using rodent endometriotic models showed that puerarin could inhibit the growth of endometriosis–like tissues as well as the expressions of endometriosis-related genes. However, no data concerning the puerarin’s effects on human eutopic/ectopic endometrium or symptoms of endometriosis patients is available at this time.

## 6. Critical Issues Concerning Studies of Phytoestrogens’ Effects on Endometriosis

### 6.1. Estrogenic or Anti-Estrogenic? A Mechanistic Dilemma of Phytoestrogens

**Figure 1:**
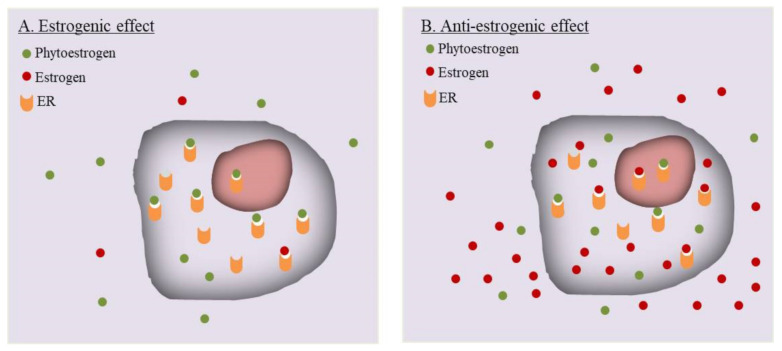
The interplay between phytoestrogens and background estrogens. (**A**). In the absence or the presence of a diminished level of estrogen, e.g., in an estrogen–free experimental system, or inside males, postmenopausal women, or pre-pubertal women, the un-liganded ER molecules are mostly available for phytoestrogen to bind. Despite their relatively low affinity to ER and low transcriptional activation potency, phytoestrogens will bind to ERs, and the consequential effects would be likely estrogenic/agonistic in this situation. (**B**). In the presence of a high level of estrogens, e.g., addition of estrogens to an in vitro experimental system, or inside the reproductive women, or under some pathological hyperestrogen conditions, phytoestrogens when reaching a sufficient concentration, will effectively compete with estrogens for ER binding. However, phytoestrogen-bound ERs have a lower transcriptional activation potency than estrogen-bound ERs. The final effects will be dependent on the ratio of phytoestrogen-bound ERs verse estrogen-bound ERs. A relatively high phytoestrogen occupancy of ERs would likely leads to an observation of anti-estrogenic/antagonist activities of phytoestrogens. While this can be cited to explain some discrepant results from different laboratories, the simplified model would be modified and further complicated by many factors, including the estrogen/phytoestrogen interactions with non-ER proteins such as SHBG, local estrogen synthesis, tissue-specific expression of ER subtypes, and post-translational modification of ER, etc.

Review of published data often leads to a paradox regarding the estrogenic or anti-estrogenic effects of phytoestrogens. Apparently inconsistent or even completely opposite results have been reported for the same type of phytoestrogen by different laboratories. While some discrepancies could be explained by the purity of the compounds used in experiments [46], others, especially those opposite results, may be caused by an intrinsic reason. The ovary, adipose and muscles produce a large amount of endogenous estrogens, and the endometrial tissue also locally synthesizes estrogens [103]. Phytoestrogens absorbed through food digestion would reach the eutopic as well as ectopic endometrium. As illustrated in Figure 1, the exogenous phytoestrogens and endogenous estrogens will competitively bind to the same ER molecules to exert their activities. The effect of a phytoestrogen compound would be largely determined by its competition with the preexisting endogenous estrogens upon binding to ER. Thus, the levels of endogenous estrogen becomes an important denominator for the actions of phytoestrogens. 

A simple example comes from in vitro studies using an ER-positive cell line. When the experiment is performed in an estrogen-free background, e.g., by applying phenol red-free medium and charcoal-absorbed serum for removal of estrogens, phytoestrogens will bind sufficiently to ERs and likely exhibit a weak estrogenic/agonist effect. In the presence of estrogens, a high concentration of phytoestrogens may compensate for their lower affinity with ERs [48], leading to a significant occupancy of ERs. However, due to the structural difference, the phytoestrogen generally have a much lower potency for transcription activation than endogenous estrogens [47,104]. It was reported that while genistein has almost the same affinity to human ERβ as E2, its concentration required to induce transcription is 10^4^ times higher than E2. The structural transformation of human ER induced by genistein is not very sufficient to promote the interaction between human ER and co-activators [50]. Their high occupancy yet weak transcription potency could explain why in many experiments phytoestrogens could display an anti-estrogenic effect. Importantly, this paradoxical phenomenon could have a significant impact on the results of in vivo studies. In animal models, even after oophorectomy, certain levels of endogenous estrogens may exist, which raises a precaution for data interpretation. Studies in human population have to face the same challenge. Males and children have low endogenous levels of endogenous estrogens, yet women at reproductive age possess relatively high whereas the menopausal women have declined, levels of estrogens. In addition, estrogen levels would vary during menstrual cycles or under other pathophysiological conditions. Conceivably, on the different estrogen backgrounds or among populations with different estrogen levels, the same dose of a phytoestrogen may exhibit different or even opposite effects.

Tsuchiya et al. compared the urinary levels of genistein and daidzein in infertility women with early endometriosis (stage I–II) and women with advanced endometriosis (stage III–Iva). It was found that high levels of urinary genistein and daidzein were negatively related to the risk of advanced endometriosis, but unrelated to the risk of early endometriosis [56]. In addition, the urinary isoflavone levels were inversely associated with the severity of endometriosis. However, contradictory results were obtained in a different setting. Mumford et al. compared the urinary levels of genistein, daidzein, O-desmethylangolensin, equol, enterodiol and enterolactone, between 189 women with endometriosis and 283 women without endometriosis, with both groups received laparoscopy or laparotomy [58]. The study also compared the levels of the same phytoestrogens between 14 women with endometriosis and 113 women without endometriosis, all received no operation. Neither comparison found a significant difference in the urinary concentrations of selected phytoestrogens between women with and without endometriosis. Since the former study concentrated on infertility women which may have abnormal estrogen levels, while the latter study focused on women with normal menstrual cycles, the different endogenous estrogen levels may partially contributed to the discrepant observations. The complicated interplay between diet phytoestrogens and endogenous estrogens should be taken into accounts during study design as well as data interpretation.

### 6.2. Dose-Dependent and Phytoestrogen Type-Specific Responses

The dose-dependent responses of phytoestrogens have been demonstrated by many experiments. Ji et al. tested an increasing concentrations (1 × 10^−13^, 1 × 10^−11^, 1 × 10^−9^, or 1 × 10^−7^ mol/L) of puerarin in the primary cultures of endometrial stromal cells from endometriosis patients and found that while all the doses inhibited the estrogen-stimulated proliferation, the concentration of 1×10^−9^ mol/L exhibited the strongest inhibition [105]. Similarly, using the same type of cell culture, Taguchi et al. observed that 10, 20 and 40 μmol/L of resveratrol inhibited the TNF-α-induced release of IL-8 in a dose-dependent manner [106]. The dose-dependent nature of phytoestrogen effects on one hand underscores a requirement for a threshold dose to achieve an efficacy, on the other hand means a necessity to empirically determine the particular effects by different concentrations of phytoestrogens.

The retrograde menstruation hypothesis has been accepted by many scholars as a pathologic mechanism of endometriosis [107]. This hypothesis emphasizes the regurgitate migration along the reproductive track and ectopic establishment of endometrial cells to the pelvic regions. The invasive growth of endometrial tissues would require local vascular expansion. Inflammation and damage of normal peritoneal tissues, which causes chronic pain and infertility, is mainly mediated by infiltration of macrophage and the release of humoral immune factors. However, the glandular, stromal, vascular endothelial, and immune cells express different types and levels of ERs, and their responses to phytoestrogens would be quite different. The cell type-specific responses to a given phytoestrogen should be taken into accounts during data interpretation as well as application of phytoestrogens for the management of endometriosis.

### 6.3. Application of Appropriate Doses for Phytoestrogen Studies

A vast range of phytoestrogen doses were used by different laboratories, and “overdosing” is often found in published studies. For example, amounts as high as 40–120 μmol/L and as low as 10–40 μmol/L of reservatrol were applied to treat human endometrial stromal cells [106,108]. For animal studies, 6 mg/day [67] and 10–25 mg/kg [69] of reservatrol were applied in mice. Supposing an average body weight of 20 g of each mouse, the latter dose can be converted to 0.2–0.5 mg/day per mouse. There is at least a 12-fold difference between the two experiments, making it difficult to compare the results. In addition, the quantitative relationship between consumption and circulatory levels of phytoestrogens has not yet established, making it difficult to compare the oral and injection doses. So far very little is known about individual variations in the metabolism, excretion, and turnover rate of phytoestrogens. These knowledge gaps need to be filled by future investigation. 

The does issue also concerns the extrapolation of animal model findings to human beings. Reagan-Shaw et al. pointed out that in the assessment of the anti-aging effects of reservatrol, “the dose used in mice could be interpreted to mean several hundreds or even thousands of liters of wine per day in human equivalent doses” [109]. They further suggested that the doses used in animal experiments should not be determined by a simple, bodyweight-based conversion, and the body surface area (BSA) normalization method could be more reasonable. In an animal studies by Rudzitis-Auth et al., 40 mg/kg of reservatrol was orally administered to mice [68]. This dose can be converted to 194.4 mg/day (3.24 mg/kg, 60 kg average weight) in human according to the BSA method as advocated by Reagan-Shaw et al. [109]. In two independent, human intervention studies by Maia et al. [76] and Mendes da Silva et al. [77], the oral doses of 30 mg/day and 40 mg/day were used, respectively. Thus, the dose used in the human intervention was approximately 5-times lower (30 mg/day and 40 mg/day verses 194.4 mg/day) than those applied in the mouse experiments. 

More and more investigators carefully choose reasonable doses in phytoestrogen studies. In a recent study on the effects of isoflavone aglycones using a mouse allotransplant endometriotic model, the authors carefully contemplated the doses for in vitro experiments and the equivalence of doses in animal studies to human intake. Two μmol/L of isoflavone aglycones was considered a reasonable plasma concentration by the authors’ calculation based on the estimated human daily intake of 0.03 g. Therefore, 0.2–20 μmol/L was applied for cell culture experiments. In addition, the authors chose to use the 0.19 g/kg/day dose in mouse experiment, which is equivalent to 0.23 g/day for human (60 kg/body) based on the metabolism difference across the species. Thus, the experimental dose in mouse was approximately 7-fold higher than the human daily oral intake (0.23/0.03), which fell in an acceptable range. Choosing a reasonable dose has a huge advantage by ensuring an extrapolation of the experimental findings to human population.

### 6.4. Limitations of Current Animal Models

The currently used mouse/rat endometriosis models are constructed essentially by intraperitoneal implantation of human eutopic/ectopic endometrial tissues or normal endometrial tissues from mouse/rat. The broad application of these rodent models has led to discovery of post-establishment mechanisms, e.g., molecular events supporting the vascularization and ectopic proliferation of endometrial glandular/stromal cells [110]. These animal models can also be applied to evaluate the therapeutic benefits of phytoestrogens for readily established endometriosis. However, the forced transplantation could not recapitulate the initial retrograde process or a natural course of human endometriosis. Human endometriosis is a chronic disease characterized by a long-term development and adaptation, and the abrupt and forced implantation of endometrial tissues does not conform to this pattern. Moreover, when athymic mice are used for heterotransplantation, theses mice suffer a major immunodeficiency, rendering them unsuitable for studying the immune responses in endometriosis. For these reasons, precautions should be applied for data interpretation when these models are used. Development of advanced animal models capable of better recapitulating the natural course of endometriosis is paramount for delineating the pathogenic events, especially those contributing to the initial or early stage of this disease.

### 6.5. Proper Design of Intervention Studies on Phytoestrogens

Endometriosis is a chronic disease that takes many years to develop and often lasts for many years since self-healing is rare. For phytoestrogens to take any effect in endometriosis patients, no matter beneficial or adverse, a long-term and sustained actions are likely required. In addition, the consumption of phytoestrogen-containing foods also follows a long-term pattern. Several long-term studies were performed to observe how phytoestrogen may affect the endometrial histology. Unfer et al. conducted a 5-year, randomized, double-blind, placebo-controlled study covering 376 postmenopausal healthy women with 150 mg of isoflavones per day [96]. The results showed that while isoflavone supplement for 5 years, but not 30 months, was associated with an increased incidence of endometrial hyperplasia. Unfortunately, most intervention trials so far have been performed in short durations, which is insufficient to properly evaluate the full potentials of phytoestrogens for this chronic disease.

Regarding the quantification methods, since phytoestrogens come from a variety of foods, the questionnaire survey only provides a rough estimation of consumption [59,60]. Also, the absorption efficiency and metabolism of these compounds is quite different among individuals, introducing further variations. The direct measurement of circulatory or urine phytoestrogen levels has its advantage [42,59,60]. However, to be relevant to endometriosis, phytoestrogens need to reach the eutopic/ectopic endometrial targets. From this point of view, local levels of phytoestrogens, e.g., in endometrial tissues, become a critical factor. The quantitative relationship among consumption, serum and urine levels, and local levels of phytoestrogens should be investigated in future studies.

## 7. Conclusions

**Figure 2:**
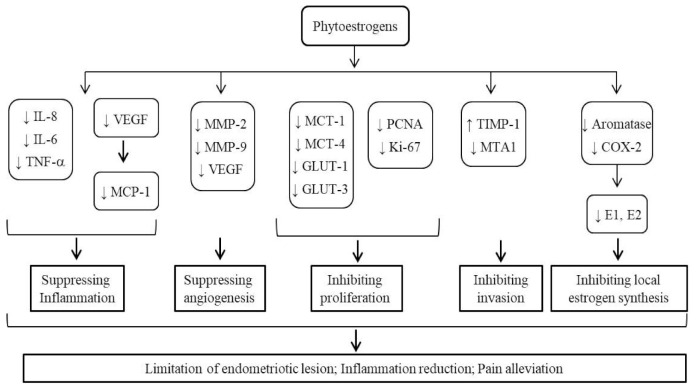
Molecular pathways and cellular effects leading to the potential therapeutic values of phytoestrogens. Positive findings in support of the application of phytoestrogens for endometriosis management were outlined. Dietary phytoestrogens are absorbed, reach the endometrial epithelial and stromal cells, bind to ER, and participate in gene regulation of a variety of endometriosis-related factors, affecting multiple processes including inflammation, angiogenesis, proliferation, invasion and local estrogen synthesis. These activities would converge to suppress the development, and alleviate the symptoms, of endometriosis. Notes: interleukin 6 (IL-6), interleukin 8 (IL-8), tumor necrosis factor alpha (TNF-α), vascular endothelial growth factor (VEGF), monocyte chemotactic protein-1 (MCP-1), matrix metalloproteinase-2 and -9 (MMP-2 and -9), monocarboxylate transporters 1 and 4 (MCT-1 and 4), glucose transporters 1 and 3 (GLUT-1 and GLUT-3), estradiol (E2), proliferating cell nuclear antigen (PCNA), Ki-67 (a proliferative marker), tissue inhibitor of metalloproteinase 1 (TIMP-1), metastasis-associated protein 1 (MTA1), cyclooxygenase-2 (COX-2), estrone (E1).

Endometriosis is by large an estrogen- and ER-dependent disease. Phytoestrogens, broadly existing in a variety of foods, have been intensively studied for their relationship with the risk of endometriosis, their potential therapeutic values, and the underlying mechanisms (Table 1). As summarized in Figure 2, phytoestrogens are able to regulate the expression of key endometriosis-related genes, and modify the pathological processes of endometriosis including inflammation, cell proliferation and invasion, angiogenesis/vasculature, and local estrogen synthesis in the endometrium. Different types of phytoestrogens with varied structures may exert divergent effects on the endometrial pathophysiology. The complicated interplay between phytoestrogens and endogenous estrogens could partially explain the seemingly discrepant or opposite actions of phytoestrogens reported by different laboratories. The current rodent models are limited in recapitulating the natural course of endometriosis, especially the initial stage of the disease. Selection of proper doses of phytoestrogens extrapolatable to human population is important for experimental design. For many clinical trials, a major limitation commonly existing is the too short period of intervention. In consideration of the accumulating effects and the consumption habit of phytoestrogen-containing foods, long-term observation is required to better evaluate the therapeutic effects of phytoestrogens. Application of objective tests for urine or blood levels of phytoestrogens will much enhance the value of an intervention study. Indeed, such information will provide a basis for pursuing the relevant mechanisms using in vitro methods and/or animal models in the follow-up studies. 

## Figures and Tables

**Table 1:** Important references and information.

	Study Subjects	Doses/Routs	Findings	References
Isoflavones, lignan, coumestrol, etc.	Infertility women (15–45 y); 78 laparoscopically confirmed endometriosis women and 78 normal controls.	Intake levels estimated based on questionnaire	Intake of phytoestrogen, isoflavones, lignan, coumestrol inversely correlated with endometriosis risk.	[55]
Genistein, daidzein	Infertility women (20–45 y); 79 with early or advance stage, 59 normal controls.	Estimated by urine concentration	Urine levels were inversely associated with the risk of advanced, not early stage endometriosis.	[56]
Genistein, daidzein, equol, etc.	Women with laparoscopy or laparotomy (18–44 y), 189 with endometriosis, 283 without; Women without operation (18–44 y), 14 with endometriosis, 113 without.	Estimated by urine concentration	Neither comparison found significant difference in urinary concentrations between groups with or without endometriosis.	[58]
Phytoestrogens from soy products	3 women with high intake of soy products.	High intake	High intake was associated with uterine bleeding and endometrial pathology; Withdrawal of soy from diet led to symptoms alleviation.	[57]
Resveratrol	Human eutopic ESCs from healthy donors; Oophorectomized mouse endometriosis model, with human endometrial tissues.	10–30 μmol/L; 6 mg/day, gavage, 10–12 and 18–20 days	Reduced invasiveness of ESCs in a concentration-dependent manner; reduced number and size of endometriotic implants.	[66]
Resveratrol	Human ectopic ESCs from ovarian endometriosis cysts; mouse endometriosis model by autotransplantation.	25 and 50 μmol/L; 25 mg/kg/day, peritoneal injection, 4 weeks.	Suppressing proliferation, migration, and invasion in ESC culture; Inhabiting ectopic tissues’ growth, MTA1 and ZEB2 expression in vivo.	[70]
Resveratrol	Human endometrial epithelial cells from endometriosis; mouse endometriotic model, autotransplantion.	25–100 μmol/L; 10 and 25 mg/kg/day, peritoneal injection, 4 weeks.	Reduced the cell proliferation and increase apoptosis; Reduced the number, volume, vascular density of endometriotic lesions.	[69]
Resveratrol	34 women (18–37 y) with severe endometriosis	400 mg, twice a day for 12–14 weeks	Decreased VEGF and TNF-α expression in eutopic endometrium.	[79]
Genistein, daidzein	Human endometrial stromal cells.	10, 20, 50, and 100 μmol/L.	Inhibited cell proliferation in dose dependent manner, decreased PRL secretion, and induced in vitro decidualization,	[85]
Isoflavone aglycones	Primary cultures from ovarian endometrioma; mouse endometriosis model, allotransplantation.	0.2–20 μmol/L, dietary supplements.	Inhibited cell proliferation; Inhibited expression of IL-6, IL-8, COX-2, aromatase, TNF-α-induced IκB phosphorylation, p65 transfer to nuclei; reduced serum glucocorticoid-regulated kinase and PGE2 levels. Decreased the number, weight, and Ki-67 activity in endometriosis-like lesions.	[88]
Isoflavones	18 postmenopausal women	7.1 ± 1.1, 65 ± 11, 132 ± 22 mg/day, oral administration, 93 days.	Have no significant influence on either the plasma levels of estrogen, androgen, gonadotropin, sex hormone binding globulin (SHBG), prolactin, insulin, cortisol, and thyroid hormone, or the vaginal cytology or endometrial biopsy.	[92]
isoflavones	Healthy postmenopausal women with intact uterus	150 mg/day for 5 years.	Increased incidence of endometrial hyperplasia.	[96]
Puerarin	Primary culture of stromal cells from ectopic endometrium of premenopausal endometriosis patients.	10^−9^ mol/L.	Reversed estrogenic activities: increasing MMP-9 expression, decreasing TIMP-1 expression, promoting invasiveness, and vascularization.	[98]
Puerarin	Primary culture of stromal cells from human ovary endometriotic cysts.	10^−9^ mol/L.	Suppressed the cell proliferation-induced by E2, partly via impeding a rapid, non-genomic, membrane-initiated ERK pathway.	[99]
Puerarin	Rat endometriotic model, autotransplantation.	60, 200 or 600 mg/kg/day, gavage, 4 weeks.	Inhibited proliferation of ectopic endometrium. Suppressed aromatase expression, reduced local estrogen biosynthesis.	[100]
pueraria flower extract	Human endometriotic 11Z and 12Z cells; mouse endometriotic model, allotransplantation.	25–100 μg/mL; 150 and 300 mg/kg/day, oral administration, 5 weeks.	Suppressed adhesion of immortalized human endometriotic cells; reduced MMP-2 and MMP-9 expression. Activated ERK1/2 pathway. Suppressed endometriotic lesion formation.	[101]

## Data Availability

Data sharing not applicable.
